# Assessment of Knowledge, Attitudes, and Factors Influencing the Selection Student of Generic Medicine

**DOI:** 10.3389/fpubh.2021.767128

**Published:** 2021-12-06

**Authors:** Mohamed N. Al-Arifi

**Affiliations:** Clinical Pharmacy Department, College of Pharmacy, King Saud University, Riyadh, Saudi Arabia

**Keywords:** generic drugs, knowledge, attitudes, factors, pharmacokinetic, bioequivalence

## Abstract

**Background:** Generic replacements for branded medicines have become a typical practice among registered pharmacists all over the world. Therefore, this study was aimed to determine the influence of the knowledge and attitudes of PharmD students and other factors on the selection of unbranded medicines.

**Methods:** A descriptive, cross-sectional study was conducted through Google Forms to collect data using self-reported questionnaires. The data was collected over a period of 3 months, from January to March 2021, among junior level pharmacy students who are currently undertaking a PharmD course at the King Saud University, College of Pharmacy, Riyadh, Saudi Arabia.

**Results:** The mean age of the students was 21.05± (SD = 1.03), majority of students 70.2% (*n* = 134) were able to define the term generic medicine, and about 65% (*n* = 123) were able to correctly define bioequivalence. More than half of the students, 56.5% (*n* = 108) lacked knowledge about the pharmacokinetic parameters of generic products. Meanwhile, the majority, 85% (*n* = 122), supported generics over branded medicines, and about 66% (*n* = 99) agreed that wider use of generic medicines would lead to less money required for the research and development of new pharmaceuticals.

**Conclusion:** Findings revealed that most pharmacy students possess sufficient knowledge of generic medicines, although knowledge in some aspects remains lacking.

## Background

With advanced healthcare, costs are on the rise, including the cost of pharmaceuticals (1, 2). Consumption of drugs has been identified to be a large factor in the increasing costs of medical services, although evidence suggested that generic replacements for branded medicines have become a typical practice among registered pharmacists all over the world (3–5). Generic drugs are pharmaceutical products that are bioequivalent to branded drugs in both physical and chemical qualities (6–8). The estimated global spending on drugs is expected to exceed USD 1.5 trillion by 2023 (9). However, reports revealed that the estimated Saudi pharmaceutical expenditures are closer to USD 8 billion (9–11). Despite the supply and demand of drugs in the market, the prevalence of communicable and non-communicable diseases have become increasingly worldwide, and Saudi Arabia is not an exemption, which in turn increases the medicine expenditure cost to the patient and health care system (9–11). According to recent estimates, ~18.5% of the population over the age of 20 is disabled, 35% is obese, and over 23% has hypertension. The Saudi Arabian healthcare burden has been noted to rise at a rapid rate in recent years, and it is expected to grow even further (9–11). However, more initiatives and investments have begun in recent years to stimulate pharmaceutical products as part of a strategic plan to produce at least 40% of all medicines locally in the long term (12, 13).

Multiple reports revealed that the use of generic drugs in comparison to branded medicines has allowed patients to save money without compromising healthcare quality (8, 13–15). The knowledge and attitudes of pharmacists were found to be influencing factors in promoting this generic substitution. Several previous studies also evaluated the knowledge and attitudes of pharmacists about prescribing generic drugs at both the national and international levels (3–6). Several previous studies measured the attitudes and perceptions of community pharmacists (3–5, 16) and other health care professionals toward generic drugs including in Saudi Arabia and other international countries (8, 13, 16). Besides this, earlier studies among practicing health care workers, including pharmacists, concluded that generic drugs are therapeutically equivalent to their brands and effectiveness was similar to branded medicine (4, 13, 17, 18). Although data among student pharmacists found negative attitudes (14). In Yemen, a previous study by Othman and Abdulghani among pharmacy students reported negative attitudes and beliefs that generic medicines are less effective than branded medicines (14). Similarly, another study by Othman and Abdulghani reported negative perceptions of generic medicines as inferior, less effective, and produced more side effects compared with branded medicines (14). Likewise, another study among health care students from Pakistan reported positive perceptions and knowledge about prescribing generic drugs (15). In Saudi Arabia, there have been many published reports examining the attitudes of practicing pharmacists. Although studies among pharmacy students remain to be lacking. As pharmacy students represent our future pharmacists, determining their views about generic prescriptions is crucial in promoting generic drug substitution. In this study, we evaluated the knowledge and attitudes of pharmacy students, as well as other factors that may affect the selection of generic drugs.

## Methods

This study used a descriptive, cross-sectional design and was conducted at the King Saud University, college of pharmacy, among PharmD students over 3 months, from January to March 2021. The data collection was carried using self-administered questionnaires through Google Forms. The inclusion criteria included were participants who were currently enrolled in the King Saud University College of Pharmacy and students of entry levels (second and third year), who were enrolled in the study. Senior students and students from other courses and other universities in Saudi Arabia were excluded from the study.

The questionnaires for this study were prepared after an extensive review of similar studies published in other countries (13, 14). The First draft of the questionnaires was reviewed by the research team comprised of a professor and the researcher. The first section had 10 questions examining the knowledge of pharmacy students on generic drugs I binary answers (Yes/No). The second section of the survey contained questions concerning attitudes, which were measured using a 5-point Likert scale ranging from “strongly agree” to “strongly disagree,” with a total of 14 items. The third section contained questions regarding possible barriers or factors that might influence the prescription of generic drugs in Saudi Arabia, adopted from a previous study (13, 14).

The questionnaires were translated into the national language using the assistance of an Arabic-speaking senior professor in the Clinical Pharmacy department and a certified Saudi Arabian translator. Before the survey questionnaires were distributed to the intended participants, a pilot study was conducted among a randomly selected group of 10 pharmacy students. The pilot study was done to test the reliability of the questionnaires. The reliability was determined using the Cronbach alpha value, which was found to be 0.71. The results of the pilot study were not included in the main study. The validated Arabic questionnaires were then used for data collection. Social media platforms were chosen as the potential medium for data collection.

The Raosoft online calculator was used to calculate the sample size for this study (http://www.raosoft.com/samplesize.html). The sample size was calculated by assuming 300 students as the population and currently registered in the university pursuing their second and third-year courses with a confidence level of 95% and a predetermined margin of error of 5%, which resulted in a sample of 169 individuals (16). We assumed that the response distribution for each question would be 50% because we are not sure what to expect the results for each question. We used 50% as the response distribution, which gave a larger sample size for this research (16).

The data were collected through online questionnaires after personally contacting the leader of the course. We created the google forms and the electronic survey link was distributed to the students. In the survey link, before answering the questionnaire, there was an introductory paragraph that talked about the objectives and importance of the study. The students were informed that their contribution was voluntary and anonymous and the students who read and agreed to the next page were redirected to research questions, considered as the consent from the student. However, this study was conducted following the guidelines of the Checklist for Reporting the Results of Internet E-Surveys (CHERRIES) (19). The ethical committees from the College of Medicine King Saud University have reviewed the questionnaires and granted permission to carry out the study. The study got institutional ethical approval from the College of Medicine, King Saud University Riyadh Saudi Arabia.

### Statistical Analysis

The data were analyzed using SPSS Version 26 (IBM, Armonk, New York, United States) for Windows. Descriptive statistics, including percentages and frequency distribution, were calculated for each variable. For the age variable, the mean values were presented.

## Results

In total, 193 PharmD students self-administered the online questionnaire. The mean age of the students was 21.05± (SD = 1.03). The knowledge of generic medications among the PharmD students is shown in Table 1. More than 70% of the students (134/193, 70.2%) were able to define the term generic medicine, and more than half (123/193, 64.4%) were able to correctly define bioequivalence. Between 89 and 65% of the surveyed students identified the requirement for the bioequivalence data, quality efficacy, and safety requirements for generic drugs, similar to the original branded products. More than half of the students (108/193, 56.5%) lacked knowledge about the pharmacokinetic parameters of generic products; however, more than 70% of the students (138/193, 72.3%) agreed that when two drug products are bioequivalent, it means that the Cmax and area under the curve (AUC) ratios estimated for each formulation can vary by 20–25%. Finally, most of the students (126/193, 66%) correctly identified that if the generic is bioequivalent to a branded medicine, it implies that it is restoratively similar.

**Table 1:** Knowledge of Saudi PharmD students on generic medicines (*n* = 193).

**Knowledge items**	**Correct (%)**	**Incorrect (%)**
1. A generic medicine is a drug that is sold under a different brand name or the drug's non-proprietary name.	134 (70.2)	57 (29.8)
2. Before they can be licensed for marketing, generic products must be bioequivalent to the innovator brand.	123 (64.4)	68 (35.6)
3. In nations that require bioequivalent data, product quality data are NOT necessary before a generic product can be registered.	170 (89)	21 (11)
4. It is thought that a generic product's efficacy, quality, and safety are comparable to the original branded product if it meets bioequivalence and product quality requirements.	123 (64.4)	68 (35.6)
5. Two pharmacological drugs are bioequivalent if they are pharmaceutically equivalent and their bioavailability is close enough that their effects can be expected to be substantially the same in terms of efficacy and safety.	112 (58.6)	79 (41.4)
6. The 90% confidence intervals for the ratio of each pharmacokinetics parameter must be within the range of 90–110% for a generic medicine to be bioequivalent to its innovator brand or other generics.	83 (43.5)	108 (56.5)
7. A generic drug is typically created without a license from the innovator business, but marketed after the patent or other exclusive rights on the original medicine have expired.	133 (69.6)	58 (30.4)
8. When two medicinal products are bioequivalent, the calculated Cmax and AUC ratios for each formulation can differ by 20–25%.	138 (72.3)	53 (27.7)
9. Where a “generic substitution” policy exists, community pharmacists are permitted to distribute a different brand of the drug, but may or may not refer the patient back to the doctor, depending on the jurisdiction/law.	94 (49.2)	97 (50.8)
10. If a generic drug is bioequivalent to a branded drug, it is also therapeutically equivalent.	126 (66)	65 (34)

Students reported that cheaper costs to patients (153/193, 80.1%); cost-effectiveness of generic medicines (137/193, 72%); availability of policies, laws, and regulations (133/193, 69.6%), and legal implications (126/193, 66%) were the most important factors influencing generic drug selection (Table 2).

**Table 2:** Possible factors influencing generic drug selection.

**Variables**	**Least important factor *n* (%)**	**Important factor** ***n* (%)**	**Neutral** ***n* (%)**
Lack of faith in generic drugs	23 (12)	85 (44.5)	83 (44.5)
Policies, laws, and regulations are readily available.	16 (8.4)	133 (69.6)	42 (22)
Consequences for law	17 (8.9)	126 (66)	48 (25.1)
Customer costs are lower.	15 (7.9)	153 (80.1)	23 (12)
No other option is available.	29 (15.2)	108 (56.5)	54 (28.3)
The appearance or nationality of the customer	115 (60.2)	29 (15.2)	47 (24.6)
Generic drugs are cost-effective.	15 (7.9)	137 (71.7)	39 (20.4)
Confidence in the product	46 (24.1)	89 (46.6)	56 (29.3)

As per our findings, the majority of students (122/193, 85%) reported (agreed/strongly agreed) that they would support generic over brand name drugs in all cases where a generic is available. More than half of the students (99/193, 66.7%) agreed that the wider use of generic medicines would mean that less money would be required for the research and development of new pharmaceuticals. Further, most students (75/193, 64%) have also agreed that the use of generic medicines would result in decreased healthcare expenditure by the government. When asked about therapeutic equivalence, most of the students (116/193, 82.7%) accepted that all products approved as generic drugs by the health authorities in the state of Saudi Arabia can be considered therapeutically equivalent to their branded counterparts; ~69% (101/193) of the students agreed that the price difference between generic and branded drugs is often so great that they feel they should dispense prescriptions of generic substitutions, especially for people who do not have prescription drug benefits in Saudi Arabia.

When asked about the requirements of generic drugs, 113/193 (86.4%) of the students agreed that health authorities should implement policies that bioequivalence data are mandatory before a generic product is marketed. More than half of the students (87/193, 60.4%) thought that pharmacists should be allowed to perform generic substitutions without consulting the prescribing physician. Slightly less than half (75/193, 48%) agreed that they should consult physicians before prescribing generics to patients; however, about 62% (92/193) of the students accepted that pharmacists should be required to consult the prescribing physician when substituting certain categories of drugs with narrow therapeutic indices. The details of the attitudes of Saudi PharmD students toward generic substitutions are described in Table 3.

**Table 3:** Attitudes on generic medicines utilization (*n* = 193).

**Variables**	**Strongly agree** ***n* (%)**	**Agree** ***n* (%)**	**Disagree** ***n* (%)**	**Strongly disagree** ***n* (%)**	**Neutral** ***n* (%)**
In all circumstances where a generic is available, I favor generic substitutes for brand-name medications.	45 (23.6)	77 (40.3)	9 (4.7)	5 (2.6)	55 (28.8)
Less money will be spent on research and development of novel drugs as generic medicines become more widely used.	32 (16.6)	67 (34.7)	33 (17.3)	15 (7.8)	44 (23)
The government of Saudi Arabia will save money on healthcare if generic drugs are used more widely.	23 (11.9)	52 (26.9)	49 (25.7)	12 (6.2)	55 (28.5)
Switching a patient from a branded to a generic treatment can have an impact on the drug's outcome.	16 (8.4)	36 (18.8)	64 (33.5)	21 (11)	54 (28.3)
Most generic products have a high rate of therapeutic failure.	19 (9.9)	43 (22.5)	47 (24.6)	20 (10.5)	62 (32.5)
All medications approved as generic drugs by Saudi Arabia's health authorities can be regarded therapeutically similar to their brand-name counterparts.	47 (24.6)	69 (36.1)	14 (7.3)	4 (2.1)	57 (29.8)
Because the price difference between generic and branded drug is frequently so large, I feel compelled to administer prescriptions with generic replacement, especially for those who do not have prescription drug coverage in Saudi Arabia.	34 (17.8)	67 (35.1)	26 (13.6)	7 (3.7)	57 (29.8)
Patients should be given a thorough explanation of why generic drugs were chosen for their therapy.	45 (23.6)	59 (30.9)	14 (7.3)	34 (17.8)	39 (20.4)
When it comes to dispensing generics, the intensity of promotional actions by medical representatives is crucial.	19 (9.9)	70 (36.6)	9 (4.7)	19 (9.9)	74 (38.7)
Bioequivalence evidence should be required before a generic product is marketed in Saudi Arabia, according to health officials.	58 (30.4)	55 (28.8)	5 (2.6)	22 (11.5)	51 (26.7)
Without consulting the prescribing physician, pharmacists should be able to make generic substitutions.	32 (16.8)	55 (28.8)	37 (19.4)	20 (10.5)	47 (24.6)
Pharmacists must consult with the prescribing physician before beginning generic substitution.	19 (9.9)	56 (29.3)	32 (16.8)	24 (12.6)	60 (31.4)
Only when substituting specific types of medications, such as those with a limited therapeutic index, should pharmacists speak with the prescribing physician.	29 (15.2)	63 (33)	23 (12)	20 (10.5)	56 (29.3)
In general, I would not provide my patients generic medications (in the future, if I became a pharmacist in KSA).	4 (2.1)	23 (12)	60 (31.4)	28 (14.7)	76 (39.8)

**Figure 1:**
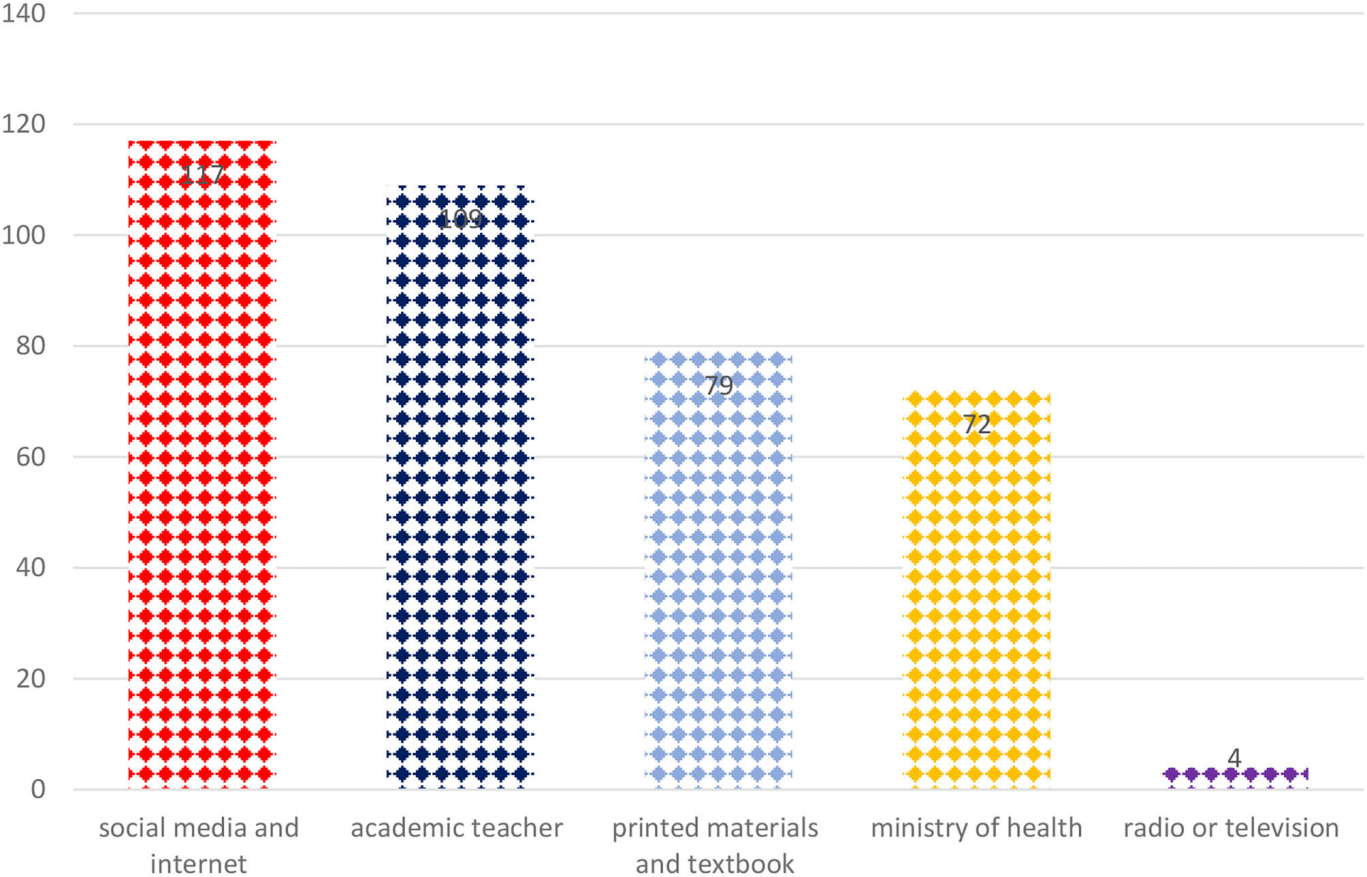
Resources about generic medicine.

The most common source of information for the generic medicine among the students was social media and the internet 117 (60%), followed by academic teacher 109 (55.9%), printed materials and textbook 79 (40.5%), ministry of health 72 (36.9%), and radio or television 4 (2.7%) (refer to Figure 1).

## Discussion

In this study, the responses of male pharmacy students were collected, but the female students were excluded. This is likely because of the Islamic prevalence of Saudi Arabia, where men are prohibited to interact with females and where coeducation is strictly prohibited. In this study, ~65% of the students agreed that generic products are similar in efficacy, quality, and safety compared to the original branded products. These findings were in line with earlier studies from Ethiopia (67%) (20), but higher than the findings of James et al. (55%) (21), Belay et al. (52.9%) (22), Toklu et al. (46.1%) (23), and Al Hussain et al. (42.2%) (24). However, our results were still lower than earlier studies by Grover et al. (70%) and Wajid et al. (72.2%) among community pharmacists (3, 25). One study among Yemeni pharmacy students from private universities revealed that students perceived generic medicines to be inferior and less effective and, thus, could lead to more side effects compared with branded medicines (14). The difference in knowledge about generic medicines might be due to the fact that the majority of respondents in the current study are currently undergraduates and entry-level students with a lack of working experience or training in pharmacy settings.

Only about 44% of the students correctly answered that pharmacokinetic parameters of generic drugs must lie within 90–110% of their branded counterparts, with a 90% CI, which is lower than the previous study among Yemeni students (55.2%) (14). These current findings suggested that Saudi students might have less understood the concept of bioequivalence and its limits for generic medicine. In this study, the majority of the students used social media as the source for generic medicine followed by academic teachers and printed materials. As evidenced from the current findings how prevalent internet use is among students as it is providing medical and pharmaceutical-related information very quickly at any time period, which might be the fact that students had good knowledge about generic drugs and substitution.

This study found that 85% of the students support generic substitution, while about 67% believe that generic medicines are cheaper, consistent with earlier studies published among pharmacists and pharmacy students. The majority (80%) of students knew the definition of generic drugs, which is similar to earlier studies published among Pakistani and Yemeni students (14, 15). This finding suggested that most students and working pharmacists were well-informed about the prescription of generic drugs, perhaps due to various hospitals and community outlet training and rotations required before their graduation. These current study results were also consistent with those of a previous study published among pharmacy and medical students from Pakistan, which reported that both pharmacy and medical students agreed that the use of generics had the capacity to reduce pharmaceutical expenses as compared to high-priced name brand medicines (15). In our study, slightly less than half of the students agreed that prescribing generics saved costs to the government as well as to the patients. According to this study, most of the students disagreed that therapeutic failure is a serious problem with most generic products, and the majority of students agreed that all pharmaceuticals approved as generic drugs by the health authorities in Saudi Arabia can be considered therapeutically equivalent to their branded counterparts. These findings also revealed that students had a positive attitude toward generic drugs, which is similar to earlier studies from different countries (15).

Earlier studies suggested that the availability of generic drugs in the pharmacy was mainly due to bonuses being offered by pharmaceutical companies, which was a potential influencing factor for prescribing generics and maximizing pharmacy profits (14, 15, 17). This earlier finding shows that pharmacists and healthcare professionals are also prone to drug promotions and, therefore, need to be trained on how to objectively evaluate drug information from the manufacturers (14, 15, 17).

In this study, students identified that having the cheapest cost to patients; cost-effectiveness of generic medicines; availability of policies, regulations, legal implications; and having no other choices were potential influencing factors in generic drug selection. However, earlier studies found that to improve the awareness and promote the prescription of generic drugs in the public healthcare system, there is a need to publicize the quality control tests of generic drugs, which were similar to the quality of branded medicines (26). Furthermore, educational interventions were most beneficial in improving the knowledge of generic drugs among students and practicing pharmacists, as reported by Almangour et al. (27). In addition to this, allocation of financial sources and good patient–healthcare professional communication can create a positive impact of generic medicines in the minds of patients and the healthcare system. Although the current had some limitations, first this study is limited to only male pharmacy students, from a single institution, therefore the results cannot be generalizable to the whole of Saudi Arabia. Second, the study included junior-level students who are currently in their second and third year of the PharmD course. The cross-sectional nature of the study, could not be able to find out the factors affecting the generic medicine knowledge. We recommend that further studies among pharmacy students with a larger sample size are needed to create awareness and to improve the knowledge toward generic medicine in Saudi Arabia and other countries are needed.

## Conclusion

This present study identifies that junior Pharm D students from a single university in Saudi Arabia had acceptable knowledge with respect to generic medicines. The matter of generic medicine cost and quality need to be made a focus for students lacking adequate knowledge in some aspects of the pharmacokinetics of generic medicines. Educational intervention and development of policies by healthcare government officials can improve the practice of generic medicine substitution in Saudi Arabia.

## Data Availability Statement

The raw data supporting the conclusions of this article will be made available by the authors, without undue reservation.

## Ethics Statement

Ethical Committees from College of Medicine, King Saud University was reviewed the questionnaires and granted permission to carry out the study, study got institutional ethical approval from College of Medicine, King Saud University, Riyadh, Saudi Arabia. The patients/participants provided their written informed consent to participate in this study.

## Author Contributions

The author confirms being the sole contributor of this work and has approved it for publication.

## Conflict of Interest

The author declares that the research was conducted in the absence of any commercial or financial relationships that could be construed as a potential conflict of interest.

## Publisher's Note

All claims expressed in this article are solely those of the authors and do not necessarily represent those of their affiliated organizations, or those of the publisher, the editors and the reviewers. Any product that may be evaluated in this article, or claim that may be made by its manufacturer, is not guaranteed or endorsed by the publisher.
